# Seven Genes Associated With Lymphatic Metastasis in Thyroid Cancer That Is Linked to Tumor Immune Cell Infiltration

**DOI:** 10.3389/fonc.2021.756246

**Published:** 2022-01-24

**Authors:** Linfeng Wu, Yuying Zhou, Yaoyao Guan, Rongyao Xiao, Jiaohao Cai, Weike Chen, Mengmeng Zheng, Kaiting Sun, Chao Chen, Guanli Huang, Xiaogang Zhang, Ziliang Qian, Shurong Shen

**Affiliations:** ^1^ Oncology and Hematology, Wenzhou Hospital of Integrated Traditional Chinese and Western Medicine, Wenzhou, China; ^2^ Thyroid Surgery, The First Affiliated Hospital of Wenzhou Medical University, Wenzhou, China; ^3^ Hongyuan Biotech, Suzhou Biobay, Suzhou, China; ^4^ Prophet Genomics Inc, San Jose, CA, United States

**Keywords:** thyroid cancer, lymphatic metastasis, immune infiltration, prognosis, THCA

## Abstract

**Objective:**

Since there are few studies exploring genes associated with lymphatic metastasis of thyroid carcinoma (THCA), this study was conducted to explore genes associated with lymphatic metastasis of THCA and to investigate the relationship with immune infiltration.

**Methods:**

Differentially expressed genes associated with THCA lymphatic metastasis were analyzed based on The Cancer Genome Atlas Program (TCGA) database; a protein-protein interaction(PPI)network was constructed to screen for pivotal genes. Based on the identified hub genes, their expression in THCA with and without lymphatic metastasis were determined. Functional enrichment analysis was performed. The correlation between the identified genes and immune cell infiltration was explored. LASSO logistic regression analysis was performed to determine the risk score of the most relevant gene constructs and multifactor COX regression analysis based on genes in the risk score formula.

**Results:**

A total of 115 genes were differentially expressed in THCA with and without lymphatic metastasis, including 28 upregulated genes and 87 downregulated genes. The PPI network identified seven hub genes (EVA1A, TIMP1, SERPINA1, FAM20A, FN1, TNC, MXRA8); the expression of all seven genes was upregulated in the group with lymphatic metastasis; Immuno-infiltration analysis showed that all seven genes were significantly positively correlated with macrophage M1 and NK cells and negatively correlated with T-cell CD4+ and myeloid dendritic cells. LASSO logistic regression analysis identified the five most relevant genes (EVA1A, SERPINA1, FN1, TNC, MXRA8), and multi-factor COX regression analysis showed EVA1A, SERPINA1 and FN1 as independent prognostic factors.

**Conclusion:**

Seven genes were associated with lymphatic metastasis of THCA and with tumor immune cell infiltration.

## Introduction

Thyroid carcinoma (THCA) is the most prevalent endocrine cancer worldwide. It usually presents as a neck mass and causes dysphagia, dysphonia or hoarseness, stridor, and dyspnea due to its occupying effect on the esophagus and trachea (1, 2). Although THCA mortality has remained relatively low and has even steadily declined in some countries, the incidence of THCA has increased significantly in recent decades (3). The rising incidence of THCA has had a significant impact on the elderly population, as adults aged 65 years and above have experienced the greatest increase in THCA incidence and the greatest change in cancer prognosis. This further leads to a greater risk of THCA treatment as well, due to lower renal clearance, increased likelihood of radioiodine overtreatment, and increased risk of arrhythmias and bone loss due to suppressive doses of thyroid hormone replacement (4).

Lymphatic metastases are usually strongly associated with reduced cancer survival (5). For most cancers, the dissemination of tumor cells through the lymphatic system is the most common route of metastasis. Lymphatic vessels surround solid tumors and promote metastasis by increasing the hyperpermeability of capillaries and the expansion of collecting vessels (6). Therefore, the presence of lymphatic metastases is an important indicator of tumor progression and a sign of worsening tumor staging. Suitable biomarkers should not only monitor disease progression and response to therapy but also identify patients at high risk of recurrence (7). With the emergence of new effective therapies, drugs have been developed to play an important role in inhibiting tumor lymphatic metastasis by targeting biomarkers and thus. For example, it has been suggested that anlotinib may prevent lymphangiogenesis and distant lymphatic metastasis in lung adenocarcinoma by inactivating VEGFR-3 phosphorylation (8). In THCA, it has been indicated that miRNA-148a, a member of endogenously expressed small non-coding RNA molecules, can inhibit cell growth and metastasis of papillary thyroid carcinoma through STAT3 and PI3K/AKT signaling pathways (9). However, the underlying mechanism of THCA development is still unknown, so a predictive biomarker is needed to determine disease progression and prognosis. The present study, however, explores genes that may affect lymphatic metastasis of THCA based on comprehensive bioinformatics analysis and investigates their relationship with immune infiltration to provide new ideas for the clinical treatment of lymphatic metastasis of THCA.

## Methods

### Data Access

The present study used the RNAseq dataset of THCA from The Cancer Genome Atlas Program (TCGA) database. We identified patients with thyroid cancer, including papillary and follicular subtypes. A total of 460 samples were included in this study, including 229 samples with no lymphatic metastases (N0) and 231 samples with lymphatic metastases (N1, N1a, N1b).

### Differences in Gene Expression With Lymphatic Metastases

The data of THCA samples from the TCGA database were divided into two groups: with lymphatic metastasis and without lymphatic metastasis. Then, the differential expression of mRNA was investigated using the Limma package of R software. When “adjusted p value < 0.05 and log2 (fold change) > 1 or log2(fold change) < −1” was defined as the threshold mRNA differential expression screening.

### Protein-Protein Interaction(PPI) Network Construction

PPI networks of lymphatic metastasis–associated genes were analyzed based on the Metascape online database (https://metascape.org/gp/#/main/step1). The hub genes were determined using the MCODE algorithm.

### Immune Infiltration Assessment

Spearman correlation was used to analyze the relationship between gene and immune cell infiltration. The horizontal coordinates represent genes, the vertical coordinates represent immune cells, and the correlation coefficients range from [-1, 1], with negative values representing a negative correlation and positive values representing a positive correlation. A statistically significant difference was indicated when p < 0.05.

### Enrichment Analysis

Gene Ontology (GO) enrichment analysis (BP: biological process; CC: cellular component; MF: molecular function) and Kyoto Encyclopedia of Genes and Genomes (KEGG) pathway analysis were performed on the screened lymphatic metastasis–associated genes using R package cluster profiles. A cut-off value of p < 0.05 was used to enrich functional categories and pathways.

### Prognostic Modeling

Seven genes significantly associated with lymphatic metastasis were subjected to LASSO regression methods to obtain the best-associated genes. Five genes associated with prognosis were identified, and a five-gene signature was constructed. The risk score was then calculated for each patient based on the regression coefficients of the genes in the signature and the corresponding expression values. Risk scores were calculated using the following formula:


Risk score=expression of Gene 1∗β1+expression of Gene 2∗β2+…+expression of Gene n∗βn

Patients were divided into high-risk and low-risk groups based on the median risk score. The Kaplan-Meier overall survival (OS) analysis was presented, followed by log-rank tests. The sensitivity and accuracy of the signature were verified by receiver operating characteristic (ROC) curves using the SurvivalROC package in R. All the above analyses were performed using the R package. Differences were indicated as statistically significant when p < 0.05.

## Results

### Analysis of Genes Differentially Expressed in Lymphatic Metastases and Non-Lymphoid Metastases

**Figure 1:**
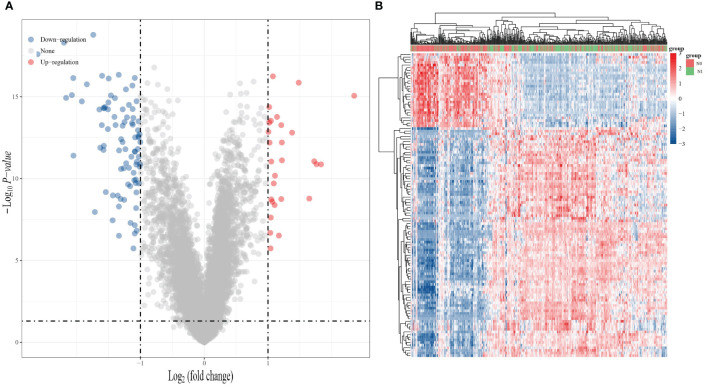
Gene expression analysis of whether THCA is lymphatic metastasis or not. **(A)** Volcano plot of differentially expressed RNA between THCA lymphatic metastasis and non-lymphoid metastasis in the TCGA dataset. **(B)** Heat map of differentially expressed RNA in the TCGA dataset. (Blue: downregulated expression; red: upregulated expression).

Based on the TCGA database, we first analyzed the genes that showed differential expression in THCA with and without lymphatic metastasis. The analysis showed that there were 115 differentially expressed genes in THCA with and without lymphatic metastasis, including 28 upregulated genes and 87 downregulated genes, as shown in the generated volcano plot (**Figure 1A**). The differential gene expression heat map demonstrates the top 100 genes with the largest differential alterations (**Figure 1B**).

### PPI Network Construction

**Figure 2:**
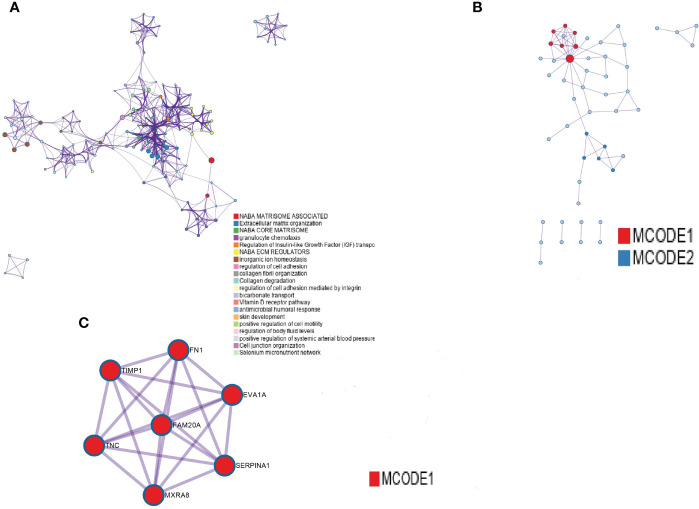
PPI network construction. **(A)** PPI networks of differentially expressed genes between the presence and absence of lymphatic metastasis. **(B)** The first seven hub genes, with nodes of higher degree shown in bright red. **(C)** The seven marked red genes.

After the analysis of the differentially expressed genes in THCA with and without lymphatic metastasis, we constructed a PPI network out of the 28 upregulated and 87 downregulated genes (**Figure 2A**)and identified seven hub genes (**Figure 2A**, marked in red): EVA1A, TIMP1, SERPINA1, FAM20A, FN1, TNC, and MXRA8 (**Figure 2B, C**).

### Expression of the Seven Hub Genes in THCA With and Without Lymphatic Metastasis

**Figure 3:**
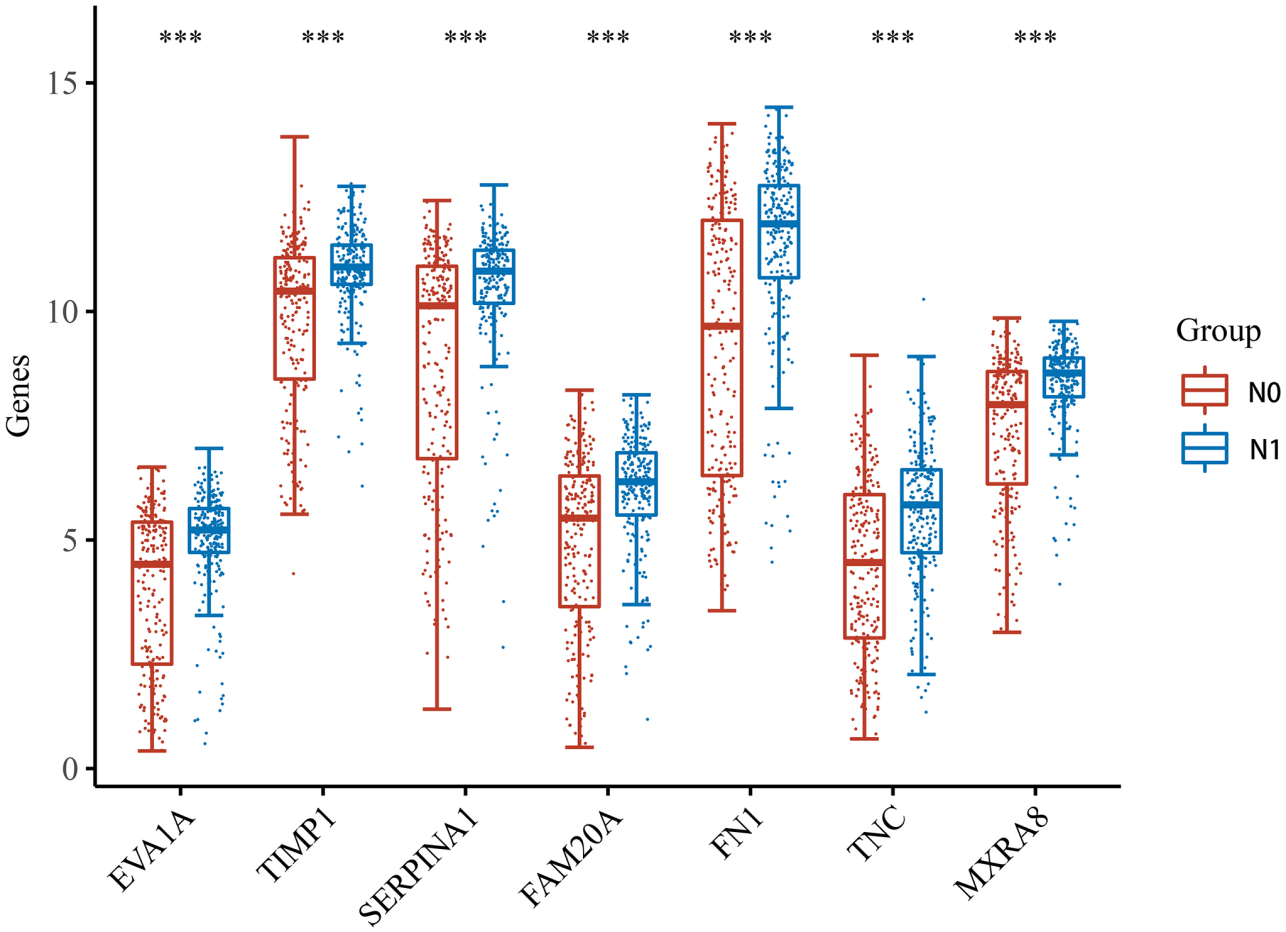
Expression of the seven hub genes in THCA with and without lymphatic metastasis. *** indicates p < 0.001 compared with N0 stage.

After the PPI network construction, we identified seven important genes (EVA1A, TIMP1, SERPINA1, FAM20A, FN1, TNC, MXRA8). Then, the THCA samples based on the TCGA dataset were divided into two groups: no lymphatic metastasis and with lymphatic metastasis. We observed the expression of these seven genes in both groups. The results of the analysis showed that the expression of the seven genes was significantly different in both THCA lymphatic metastasis and non-lymphoid metastasis, with upregulated expression in the group with lymphatic metastasis (**Figure 3**).

### GO Analysis and KEGG Analysis

**Figure 4:**
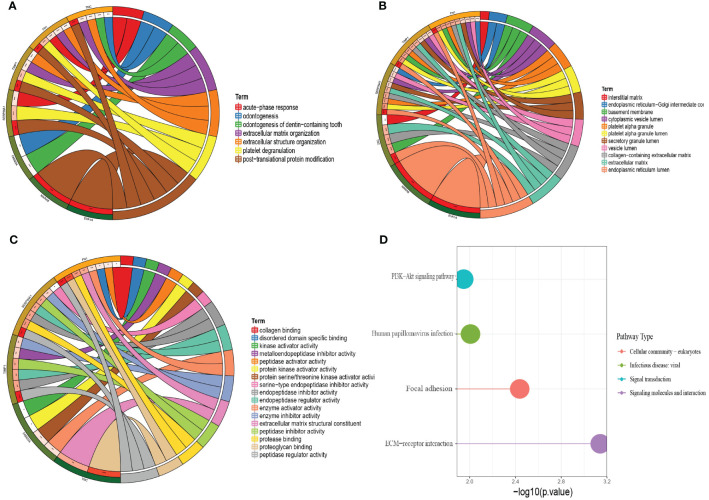
GO analysis and KEGG analysis of the seven hub genes. **(A)** Circle plot of BP analysis. **(B)** Circle plot of CC analysis. **(C)** Circle plot of MF analysis. **(D)** KEGG analysis.

To understand the molecular mechanisms of lymphatic metastasis–related genes, we performed an enrichment analysis on these seven genes. The results of the GO analysis (**Figure 4A–C**) showed that the seven genes have important roles in dentinogenesis, formation of dentin-containing teeth, acute phase response, extracellular structural organization, and extracellular matrix organization. The results of the KEGG analysis showed that the seven genes have important roles in the PI3K-Akt signaling pathway, human papillomavirus infection, local adhesions, and ECM-receptor interactions (**Figure 4D**).

### Relationship Between the Seven Hub Genes in THCA and Immune Infiltration

**Figure 5:**
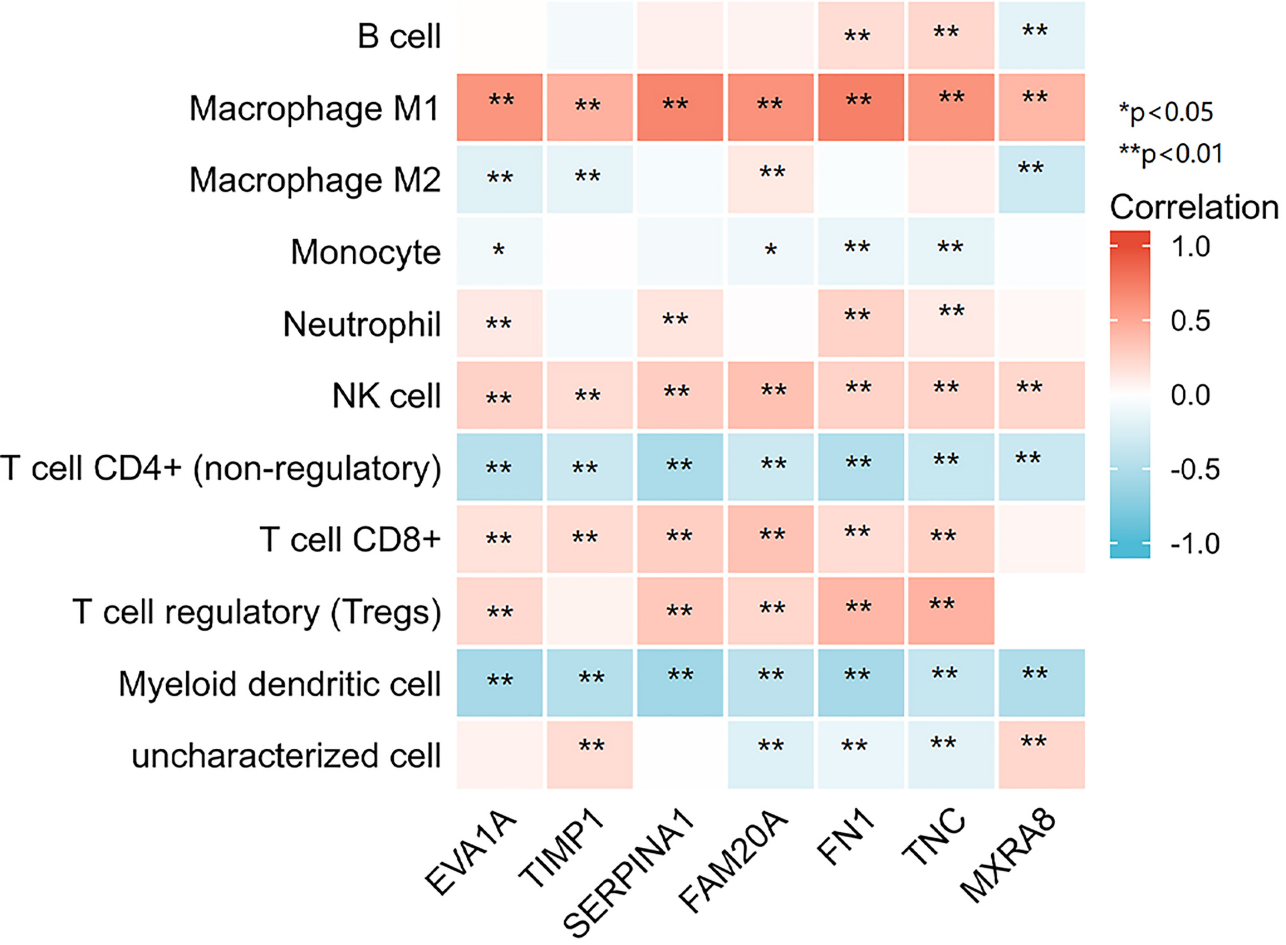
7 genes respectively in relation to the level of immune cell infiltration. *p < 0.05, **p < 0.01.

After the enrichment analysis, we found that genes have an important role in the acute phase response. It is known that acute phase responses are the body’s response to tissue damage, and acute phase responses may stimulate the initiation of the immune response (10, 11). Thus, we analyzed the relationship between the seven genes in THCA and immune infiltration. The analysis showed that all seven genes were significantly positively correlated with macrophage M1 and NK cells, and negatively correlated with T-cell CD4+ and myeloid dendritic cells, suggesting that these genes have an important role in the immune infiltration of THCA (**Figure 5**).

### Prognostic Modeling

**Figure 6:**
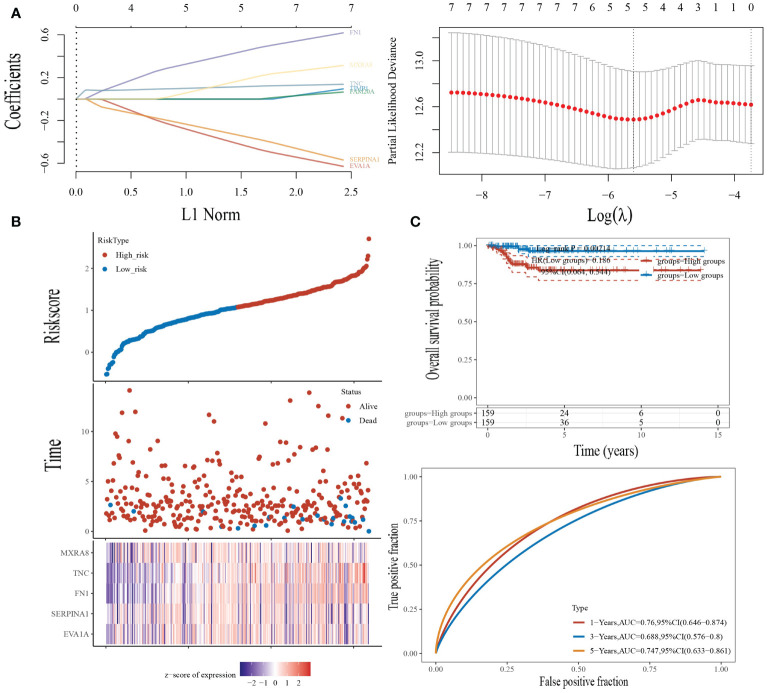
Construction of the prognostic risk model. **(A)** LASSO analysis of related genes. **(B)** Risk scores, survival time, and survival status in the TCGA dataset. Top: scatterplot of risk scores from low to high; middle: scatterplot distribution of survival time and survival status corresponding to risk scores of different samples; bottom: heat map of gene expression in the prognostic model. **(C)** Top: K-M curves for high-risk patients and low-risk patients; bottom: ROC curves for one, three, and five years for this risk model.

We performed a LASSO logistic regression analysis on the gene expression matrices of seven patients from the THCA cohort, using the presence or absence of lymph node metastasis to finalize the risk scores for the five most relevant gene constructs (**Figure 6A, B**).

The risk score was calculated by the following formula:


Riskscore=(−0.3537)∗EVA1A+(−0.2885)∗SERPINA1+(0.3839)∗FN1+(0.1075)∗TNC+(0.116)∗MXRA8

We then looked at the distribution of KM survival curves for this risk model in the TCGA dataset, which showed a significantly lower survival status for high-risk patients than for patients in the low-risk group (HR=0.186, p=0.002), as well as AUC values of 0.76, 0.688, and 0.747 for the ROC curves of this model at one, three, and five years, respectively (**Figure 6C**).

**Figure 7:**
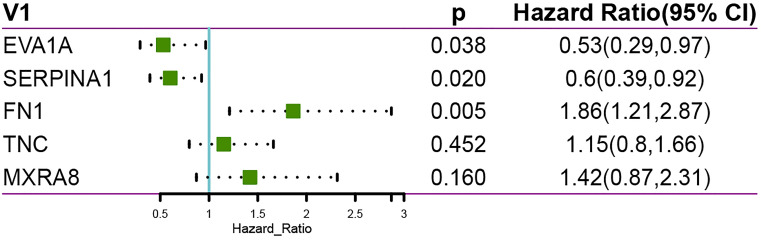
Multi-factor COX regression analysis showing EVA1A, SERPINA1 and FN1 as independent prognostic factors.

In addition, we performed a multifactorial COX regression analysis for the five genes in the risk-based scoring formula, showing that EVA1A、SERPINA1and FN1 as independent prognostic factors (**Figure 7**).

## Discussion

Tumor metastasis refers to the spread of cancer cells from the primary tumor to the circulatory system and their colonization of distant organs. It generally includes both hematogenous and lymphatic metastases and is one of the most critical aspects of tumor progression, which can lead to approximately 90% of cancer-related deaths (12). During metastasis, the lymph nodes near the primary tumor are usually the most common site of cancer cell dissemination (13). In most tumors, lymphatic metastasis is directly associated with distant recurrence and overall survival (14). Therefore, it is necessary to find biomarkers that can predict the phenomenon of early metastasis. In the present study, we detected 115 gene expression differences, 28 upregulated genes, and 87 downregulated genes in two groups of samples with and without lymphatic metastasis in THCA. We then constructed a PPI network of these genes and screened a total of seven hub genes: EVA1A, TIMP1, SERPINA1, FAM20A, FN1, TNC, and MXRA8.

EVA1A is a protein-encoding gene involved in autophagy and apoptosis-induced cell death (15). EVA1A is expressed in a cell- and tissue-specific manner and is significantly downregulated in many types of human tumors (16). TIMP1 is a secreted protein and an endogenous inhibitor of MMP9 (17). TIMP1 inhibits the protein hydrolytic activity of MMP and plays a role in the balance of matrix remodeling during extracellular matrix degradation, which has an important role in tumor invasion and metastasis (18). In colorectal cancer, increased IMP1 activity may be associated with the metastasis of colorectal cancer (19). SERPINA1, also known as α-1-antitrypsin (AAT), is a protease inhibitor constitutively released from hepatocytes. It is primarily active at sites of inflammation and usually protects healthy cells near inflamed tissue (20). Fam20A is a secretory pathway pseudokinase that forms a functional complex with Fam20C and metastasizes to increase the activity of Fam20C on secreted substrates, including enamel matrix proteins (21). FN1 is a member of the FN family, and its expression in human malignancies and has a key role in tumorigenesis and tumor progression (22). TNC is a key tendonogenic protein in the extracellular matrix glycoprotein family that often exhibits reduced expression in normal adult tissues but exhibits increased expression during embryonic development, tumor, injury repair, and inflammation. TNC has also been identified as a potentially important indicator of disease severity (23). MXRA8 is a protein that is expressed in adhesion molecules found in epithelial cells, bone marrow cells, and mesenchymal cells (24). It is also a receptor for several arthritogenic metaviruses and has been suggested as a possible drug target for infections and diseases caused by these metaviruses (25). In the present study, several genes, EVA1A, TIMP1, SERPINA1, FAM20A, FN1, TNC, and MXRA8, were found to be significantly upregulated in THCA with lymphatic metastasis. Subsequent prognostic analysis showed that only EVA1A had prognostic significance in THCA with or without lymphatic metastasis.

The lymphatic system can act as an interface between innate and adaptive immunity and can actively communicate and sense inflammatory stimuli from the periphery (26). Lymph nodes are also common sites of tumor metastasis, and cancer cells in lymph nodes can shape their interactions with the host immune system by controlling the infiltration and reactivity of immune cells (27). This could suggest that lymphatic metastasis may be closely linked to the infiltration of immune cells. There are previous findings that suggest that cancer-associated fibroblasts (CAFs) are associated with macrophage infiltration in triple-negative breast cancer patients. CAFs may play an important role in shaping the tumor immunosuppressive microenvironment by regulating the pro-tumor phenotype of macrophages (28). Previous literature has also stated that increased lymphatic vessel density in melanoma is associated with increased CD8^+^ T cell infiltration, and it has been proposed that lymphatic activation may promote the accumulation of CD8^+^ T cells around and within tumors (29). The results of our enrichment analysis also showed a possible association between the seven hub genes and immunity. Therefore, the present study also analyzed the correlation between these genes and the level of THCA immune cell infiltration. The analysis showed that all seven genes were significantly positively correlated with macrophage M1 and NK cells and negatively correlated with T-cell CD4+ and myeloid dendritic cells. It has been shown that TIMP-1 levels are elevated in chronic obstructive pulmonary disease and that its elevated levels can lead to increased neutrophil numbers and decreased lung function (30). It has also been shown that SERPINA1 has immunosuppressive effects and that with an increase in SERPINA1, the body loses immune surveillance against mutant cells and induced tumors (31). Although there are no definitive studies indicating their role in thyroid cancer on immune cell infiltration, changes in their levels have been shown to have an impact on the level of immune cells and thus on the progression of the disease. Similar studies have shown that dendritic cells and neutrophils, in papillary thyroid cancer, are strongly associated with histological subtype, mutational status, T-staging and lymph node metastasis (32).This further proves that immune cell infiltration can promote lymphatic metastasis of thyroid cancer. Combined with this study, it can be seen that this process is closely related to these seven key genes, but the specific mechanism remains to be studied.

Finally, we performed a LASSO logistic regression analysis of the gene expression matrix of seven of the THCA cohort from the TCGA database by the presence or absence of lymph node metastasis to finalize the risk scores constructed for the five most relevant genes (EVA1A, SERPINA1, FN1, TNC, MXRA8). In addition, multivariate Cox regression analysis based on the five genes in the risk score formula showed that eva1a, serpina1 and FN1 were independent prognostic factors. It can be explained that eva1a, serpina1 and FN1 are closely related to the prognosis of THCA lymphatic metastasis.

It should be acknowledged that our study has some unavoidable limitations. TCGA is a regularly updated public database, but the sample size and data volume are limited, and the clinicopathological information is not comprehensive. This may lead to some potential errors or biases. More data should be included in the future to improve the model. Given that the study is based on bioinformatics analysis, validation from *in vivo* and *in vitro* experiments is lacking. We will also continue the study.

In summary, Our results showed that seven genes were associated with lymphatic metastasis in THCA, and all seven genes were significantly positively correlated with macrophage M1 and NK cells, and negatively correlated with T-cell CD4+ and myeloid dendritic cells. This suggests that these seven genes may promote lymphatic metastasis of THCA through immune cell infiltration. In addition, EVA1A, SERPINA1 and FN1 were strongly associated with the prognosis of THCA lymphatic metastasis. This provides a potential direction for immunotherapy to stop the progression of THCA.

## Data Availability Statement

The original contributions presented in the study are included in the article/supplementary material. Further inquiries can be directed to the corresponding author.

## Author Contributions

We contributed equally for this work. All authors contributed to the article and approved the submitted version.

## Conflict of Interest

Authors XZ and ZQ were employed by Suzhou Biobay and Prophet Genomics Inc.

The remaining authors declare that the research was conducted in the absence of any commercial or financial relationships that could be construed as a potential conflict of interest.

## Publisher’s Note

All claims expressed in this article are solely those of the authors and do not necessarily represent those of their affiliated organizations, or those of the publisher, the editors and the reviewers. Any product that may be evaluated in this article, or claim that may be made by its manufacturer, is not guaranteed or endorsed by the publisher.
